# Somatosensory Event-Related Potential as an Electrophysiological Correlate of Endogenous Spatial Tactile Attention: Prospects for Electrotactile Brain-Computer Interface for Sensory Training

**DOI:** 10.3390/brainsci13050766

**Published:** 2023-05-05

**Authors:** Marija Novičić, Andrej M. Savić

**Affiliations:** School of Electrical Engineering, University of Belgrade, Bulevar Kralja Aleksandra 73, 11120 Belgrade, Serbia

**Keywords:** somatosensory event-related potentials (ERP), tactile attention task, spatial electrotactile attention, somatosensory evoked potentials, electrotactile stimulation, somatosensory training, brain-computer interface (BCI)

## Abstract

Tactile attention tasks are used in the diagnosis and treatment of neurological and sensory processing disorders, while somatosensory event-related potentials (ERP) measured by electroencephalography (EEG) are used as neural correlates of attention processes. Brain-computer interface (BCI) technology provides an opportunity for the training of mental task execution via providing online feedback based on ERP measures. Our recent work introduced a novel electrotactile BCI for sensory training, based on somatosensory ERP; however, no previous studies have addressed specific somatosensory ERP morphological features as measures of sustained endogenous spatial tactile attention in the context of BCI control. Here we show the morphology of somatosensory ERP responses induced by a novel task introduced within our electrotactile BCI platform i.e., the sustained endogenous spatial electrotactile attention task. By applying pulsed electrical stimuli to the two proximal stimulation hotspots at the user’s forearm, stimulating sequentially the mixed branches of radial and median nerves with equal probability of stimuli occurrence, we successfully recorded somatosensory ERPs for both stimulation locations, in the attended and unattended conditions. Waveforms of somatosensory ERP responses for both mixed nerve branches showed similar morphology in line with previous reports on somatosensory ERP components obtained by stimulation of exclusively sensory nerves. Moreover, we found statistically significant increases in ERP amplitude on several components, at both stimulation hotspots, while sustained endogenous spatial electrotactile attention task is performed. Our results revealed the existence of general ERP windows of interest and signal features that can be used to detect sustained endogenous tactile attention and classify between spatial attention locations in 11 healthy subjects. The current results show that features of N140, P3a and P3b somatosensory ERP components are the most prominent global markers of sustained spatial electrotactile attention, over all subjects, within our novel electrotactile BCI task/paradigm, and this work proposes the features of those components as markers of sustained endogenous spatial tactile attention in online BCI control. Immediate implications of this work are the possible improvement of online BCI control within our novel electrotactile BCI system, while these finding can be used for other tactile BCI applications in the diagnosis and treatment of neurological disorders by employing mixed nerve somatosensory ERPs and sustained endogenous electrotactile attention task as control paradigms.

## 1. Introduction

Selective attention allows the extraction of behaviorally relevant information while ignoring distracting stimuli that compete for the central nervous system resources. Exogenous attention is typically induced by presenting an abrupt stimulus that directs attention without intention, while endogenous attention can be voluntarily directed to a specific event or spatial location to select the most relevant information for current goals [1].

A tactile attention task is a cognitive task that involves focusing attention on tactile sensations. The participant is typically presented with a series of tactile stimuli, such as vibrations, pressure, or electric pulses and is instructed to respond to specific features of the stimuli, such as their intensity, duration, or location on the body, while the effects of attention are measured by comparing responses to sensory stimuli, when they are attended vs. unattended [2]. Moreover, sustained attention to a body location results in enhanced processing of the tactile stimuli presented at that location compared to another unattended location [2].

Such tactile attention tasks are often used in research to study the neural mechanisms underlying tactile perception and attention, as well as in clinical settings to assess sensory processing disorders and other neurological conditions [3]. Tactile attention tasks are used in the diagnosis and treatment of neurological and sensory processing disorders. Tactile dysfunction is common in neurological disorders such as stroke, multiple sclerosis, Parkinson’s disease, complex regional pain syndrome and phantom limb pain. Abnormalities of tactile processing have been reported also in neurodevelopmental disorders such as autism spectrum disorder and attention-deficit/hyperactivity disorder (ADHD) [4,5,6]. 

In addition to the assessment of sensory processing, tactile attention tasks can be used for training and improvement of sensory functions, though perceptual learning mechanisms, referring to the improvement in sensory processing and perception because of experience and practice [7]. Tactile attention tasks can be used in educational settings to improve students’ sensory awareness and attentional skills [8,9], to train individuals in specific skills, such as fine motor skills or braille reading [10] and treat sensorimotor deficits in neurological disorders such as stroke [11]. Clinical work specifically, including therapeutic approaches in stroke patients participating in proprioceptive training based on tactile attention tasks, revealed altered cortical activity in both somatosensory and sensorimotor processing areas, resulting in improved sensory function as well as movement accuracy, indicating that sensory learning transfers to motor function [12,13]. Therefore, selective tactile attention training, through repeated execution of tactile attention tasks, can be a useful tool for continuous assessment and training of sensory functions.

Somatosensory event-related potentials (sERP) measured with EEG during selective attention tasks are a useful tool for exploring the spatio-temporal dynamics of neural processes involved in tactile attention [14]. Moreover, event-related potentials (ERP) are commonly used control signals in brain-computer interface (BCI) systems [15]. Such systems enable providing online feedback to the user on their task-associated neural activity.

In the context of tactile attention task training, BCI can detect changes in ERPs associated with user’s tactile attention, and provide online feedback on their performance, (i.e., specific features of neural correlates of tactile attention), which may be a useful tool for validating the immediate effects of task executions and improving the training effectiveness through the user’s online modulation of task execution, towards generating desired electrophysiological output. These real-time modulations of task execution are enabled by closing the loop via online feedback provided by the BCI device [16].

We have previously designed and presented an electrotactile BCI system that enables online detection of tactile attention, directed towards one of the two proximal stimulation locations on the user’s forearm [17]. However, the presentation and analysis of sERP morphology and topographic differences over the sensorimotor cortex during this task wasn’t previously conducted. Understanding the underlying neurophysiological mechanisms governing the changes in BCI control signals induced by endogenous spatial tactile attention can contribute to designing better feature extraction methods and improving BCI performance. Moreover, knowledge on the basic neurophysiological mechanisms, underpinning the BCI control, may also improve the effectiveness of the BCI application, especially in the context of rehabilitation or cognitive/motor skill training [18].

In this paper we present an analysis of sERP responses induced within a novel paradigm for endogenous spatial tactile attention training, particularly designed for assessing the immediate effects of the sustained spatial tactile attention task. The aim of this work is to present the basic morphology of electrically induced somatosensory ERPs derived from EEG channels placed over the sensorimotor cortex and to explore the effect of endogenous spatial tactile attention on sERP morphological features.

We have not identified other previous studies that explore the morphology of somatosensory ERPs induced by electrotactile stimuli (coupled with sustained spatial tactile attention task), delivered to the mixed branches of the median and radial nerves of the forearm, especially in the context of the prospective BCI control for somatosensory training. Therefore, this study aims to contribute to the existing knowledge on the neural correlates of endogenous spatial tactile attention, but also present evidence about basic electrophysiological signal features that could be further utilized to improve sERP based electrotactile BCI control.

This is also the first study to present the changes in sERP induced by sustained spatial tactile attention to one of two stimulation hotspots with equal (50%) probability of stimulus occurrence. Therefore, our study aimed to isolate and present the somatosensory mismatch response (within the sERP waveform morphology) which corresponds predominantly to the endogenous spatial tactile attention, while it is independent of target/distractor probability, unlike in a classical oddball paradigm with rare and frequent stimuli (directing exogenous attention towards an oddball stimuli), most often used in ERP-based BCIs (for review see [19,20]).

## 2. Materials and Methods

### 2.1. Participants

This study was performed on 15 healthy (9 male and 6 female, average age 25.6 ± 2.85) subjects without history of neurological disorders and with normal or corrected to normal vision. Volunteers were recruited among the students and employees of the University of Belgrade. The subjects had no previous experience with EEG recordings and were completely naive to the spatial tactile attention task they executed during tests. All the participants were informed about study procedures and signed a written informed consent form before participation. The study was approved by the local ethic committee and is in accordance with ethical guidelines defined by the Declaration of Helsinki.

### 2.2. Apparatus and Instrumentation

The EEG data was recorded using the g.USBamp amplifier (g.tec GmbH, Austria). Six active electrodes (g.GAMMAcap2 connected to g.GAMMAbox, g.tec GmbH, Austria) were positioned at standard 10–20 locations: C3, Cz, C4, CP3, P3 and Fp1. The ground electrode was placed at the AFz location, and the reference electrode was placed on the left earlobe. The Fp1 channel was used to detect ocular artifacts i.e., blinks, since eye movements, and were controlled during the tests. The sampling rate was set to 1200 Hz. The amplifier was configured to use embedded Notch filtering with a cut-off frequency of 50 Hz. 

Electrical stimulation was delivered by an eight-channel current-controlled electrical stimulator, MOTIMOVE (3F—Fit Fabricando Faber, Serbia). This stimulator is fully programmable with independent control of stimulation parameters (pulse width, pulse amplitude, and stimulation frequency) for each channel, by sending commands from the PC via USB port in real time. The stimulator is battery powered and fully isolated from the main power supply. For this study, two stimulation channels were used, comprised of two active electrodes of 1 cm diameter placed on the stimulated locations and a common indifferent electrode of 2.5 cm diameter.

### 2.3. Protocol

The subjects were seated in a chair in front of a computer monitor at approximately 1 m. Preparation for the tests involved setup of the EEG and electrical stimulation. For signal acquisition and stimulation control, a graphical user interface was developed in a MATLAB programming environment (Math-Works Inc., Natick, MA, USA), through which the experimenter inspected the EEG quality and controlled the electrical stimulation settings. Two active stimulation electrodes were placed on the dorsal surface (D stimulation location) and volar surface (V stimulation location) of the right forearm, over the extensor carpi radialis longus muscle, and flexor carpi radialis muscle, respectively. A common indifferent electrode was placed on the volar aspect of the right wrist. Electrical stimulation setup and fine-tuning was already described in detail in our previous publication [17]. 

Motor threshold was obtained for both electrical stimulation channels separately by increasing the pulse amplitude starting from 5 mA with 1 mA steps. Stimulation was increased over the motor threshold to inspect the muscle activation associated with each stimulation location. If the stimulation didn’t selectively activate the target muscles, stimulation electrodes were manually repositioned until selective activation of the flexor carpi radialis and extensor carpi radialis longus was successfully obtained. When the expected muscle twitch responses were observed at each location, the stimulation amplitude was reduced by 1 mA below the motor threshold. Next, the subjective stimulation sensations at two stimulation hotspots were balanced by decreasing the stimulation amplitude at the channel which induced the stronger sensory response reported by the subject. The goal was to achieve the most similar subjective sensation produced by electrical stimulation at both sites. 

The experiment consisted of 30 blocks. In each block, 60 stimuli were delivered sequentially in a randomized order to locations D and V with a 750 ms interstimulus interval. The subjects were instructed to perform a sustained spatial tactile attention task by silently counting the stimuli delivered to only one target location. The target location (D or V) alternately switched between the blocks, while the staring target location was randomized over subjects. In short pauses between the blocks the subject reported the counted number of trials and verified the attended location. Then the experimenter announced the next location to attend, instructed the subject to fix the gaze to a fixation cross on the screen, limit all movements, and initiated the next block. 

The experiment comprised of two different attention tasks: sustained attention, i.e., counting the stimuli delivered at the dorsal location (AD), and sustained attention at the volar location (AV). However, during each attention task the stimuli were delivered to both locations which results in four possible of sERP waveforms responses (conditions):

1. Attending stimuli delivered to D location, while D location was being stimulated: ADSD—attending D, stimulated D,
2. Attending stimuli delivered to D location, while V location was being stimulated: ADSV—attending D, stimulated V,
3. Attending stimuli delivered to V location, while V location was being stimulated: AVSV—attending V, stimulated V, and
4. Attending stimuli delivered to V location, while D location was being stimulated: AVSD—attending V, stimulated D.

### 2.4. ERP Processing

Offline EEG processing was implemented in a MATLAB programming environment using custom scripts for signal processing and sERP extraction. Bandpass filtering using the 2nd order zero-phased Butterworth filter was applied. Five EEG channels were filtered between 0.1 and 25 Hz and the Fp1 channel was filtered between 1 and 10 Hz. The EEG was segmented on single trial 700 ms epochs containing a 100 ms prestimulus baseline and 600 ms poststimulus interval. All epochs were baseline corrected by subtracting from the poststimulus interval the median value of baseline for each channel. Epochs containing blinks and other high amplitude artifacts were rejected by applying the threshold of 80 *µV* on filtered Fp1 channels for identifying blink artifacts, and the threshold of 50 *µV* on an absolute value on all EEG channels. Noise-free epochs were divided into four sERP responses associated with combinations of stimulus location and attention focus (ADSD, ADSV, AVSV, AVSD conditions). Finally, mean sERP responses associated with each condition were extracted by averaging all noise-free single trials per condition. The total number of trials per experimental condition per subject was 450, while the average number of rejected trials was 39.17 ± 34.9 per individual subject. For further statistical comparison of sERP peaks, latencies, and areas under the sERP voltage curves, between conditions, we applied the following methods [21]. Upon visual inspection of grand-average sERP curves over all subjects, we identified electrophysiologically relevant windows in which distinct sERP components were identified, based on the previous knowledge on sERP components present during the stimulation of upper limb sensory nerves [14,22,23,24]. After identifying search windows of interest, local maximum or minimum polarity was found (in microvolts—*µV*) for each subject and experimental condition, dependent on the sERP component. Latency in milliseconds (*ms*) of each component (maximum or minimum) was calculated also. Area under the rectified sERP voltage curve (in ms⋅μV units) within each selected window was calculated using the trapezoidal rule.

### 2.5. Statistical Analysis

The aim of the statistical analysis was to assess the influence of the sustained spatial attention task on sERP morphology for the same electrical stimulation hotspot. Therefore, our analysis was constrained to the comparison of conditions associated with the same stimulation location (ADSD vs. AVSD condition, or AVSV vs. ADSV condition).

We conducted analysis of sERP amplitude peaks, peak latencies, and areas under the voltage curves between associated conditions. Peaks, latencies, and areas were extracted from several time windows of the poststimulus interval, while the window selection was explained in more detail in the following sections. For the sERP peak, latency, and area under the voltage curve analysis, we extracted the maximum amplitude, minimum amplitude, the latency of each peak, and area in predetermined subintervals (windows) of the poststimulus sERP waveforms. Wilcoxon signed rank test was used to determine significant difference (*p* < 0.05) in extracted values, for each channel, over all subjects, between conditions associated with the same stimulation location.

We also conducted a deeper exploratory analysis of the sERP amplitude at different time instants of the poststimulus interval [25]. The goal was to identify the time instants (for each EEG channel) corresponding to statistically significant differences between conditions associated with the same stimulation hotspot. The Wilcoxson signed-rank test was used for the assessment of significant difference (*p* < 0.05) between sERP waveform amplitudes for each time point (signal sample) of each EEG channel, over all subjects.

Additionally, to eliminate errors due to multiple comparisons while maintaining a relevant statistical output, in line with electrophysiological expectations [25], we have kept only the intervals of statistical significance of at least 10 ms duration, or longer. With the sampling rate of 1200 Hz, this translates to the requirement that the interval of significance is considered valid only if 12 consecutive signal samples are significantly different.

## 3. Results

Data from four subjects was rejected from analysis due to the following reasons. One subject’s data was contaminated with electrical stimulation artifacts which contributed to a high number of noisy epochs while three subjects were unsuccessful in the sustained attention task. These subjects had a high incidence in incorrect reported counts (in multiple blocks) and had problems in sustaining attention at the instructed location which was reported between the blocks. Namely, subjects reported attending to the different location then requested, incorrect counts due to counting stimuli at both locations or failing to count stimuli at target locations. The present results are extracted from 11 remaining subjects (8 male and 3 female, average age 25.54 ± 3.01).

**Figure 1:**
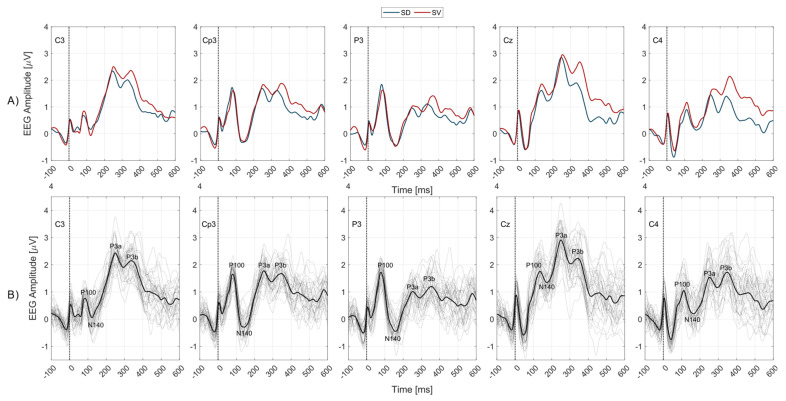
Grand average sERP waveforms over all subjects for 5 EEG channels. Zero on the time axes marks the stimulus onset (vertical black dotted lines). EEG channel labels are presented within each subplot. (**A**) In subplots of the top row, blue lines represent sERP responses to D location stimuli and red lines to V location stimuli independent of the attention task (attended and unattended trials pooled and averaged together), as indicated in legend included in the subplots title. (**B**) In subplots of the bottom row, gray lines mark the individual sERP responses of a single subject (4 lines per subject, single trials of all conditions randomized and averaged), while the black line shows the grand average sERP over all subjects and conditions. Labels of four distinct ERP components, P100, N140, P3a, and P3b are included in each subplot next to the identified peak.

Figure 1A presents sERP morphology for stimulation locations D and V independent of the tactile attention task, i.e., with conditions ADSD and AVSD averaged together, over all subjects, presented with a blue curve, and conditions AVSV and ADSV averaged together and presented with a red curve. The black line in Figure 1B shows the grand average sERP over all subjects and conditions. Waveforms presented in Figure 1A display similar morphology independent of the stimulation location with same sERP components present for both stimulation hotspots. 

Based on the grand average sERP waveform morphology (Figure 1B), over all subjects, for all channels and conditions, we have identified the following sERP peaks:

1. Positive peak marked as P100, at latencies ranging from 75 to 135 ms over different EEG channels,
2. Negative peak marked as N140, at latencies ranging from 115 to 165 ms over different EEG channels,
3. First positive local maximum within a larger window between 200 and 400 ms, termed P3a, and
4. Second positive local maximum within a larger window between 200 and 400 ms, termed P3b.

**Figure 2:**
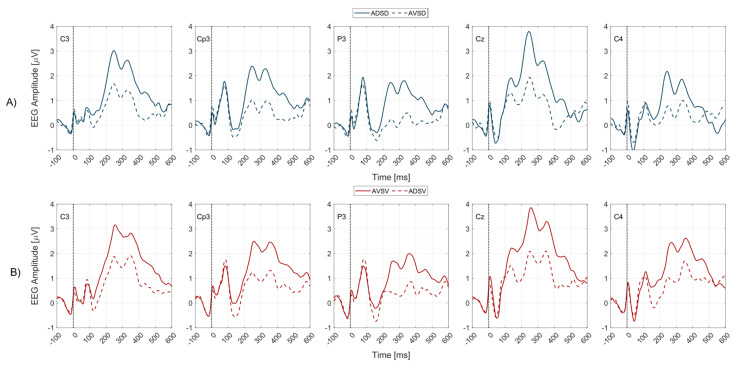
Grand average sERP waveforms over all subjects for 5 EEG channels. Zero on the time axes marks the stimulus onset (vertical black dotted lines). EEG channel labels are presented within each subplot. The solid lines (solid blue or red) represent the attended condition while the colored dashed lines (dashed blue or red) represent the unattended condition. The blue lines represent sERP responses to D location stimuli and red lines to V location stimuli. (**A**) Full blue lines are sERP responses associated with ADSD condition while the dashed blue lines are sERP responses associated with AVSD condition for all EEG channels. Therefore, the top row of subplots (**A**) represents sERP responses associated with mixed radial nerve stimulation when the stimuli were attended (solid blue lines) vs. unattended (dashed blue lines). (**B**) Full red lines are sERP responses associated with AVSV condition while the dashed red lines are sERP responses associated with ADSV condition, for all EEG channels. Therefore, bottom row of subplots (**B**) represents sERP responses associated with mixed median nerve stimulation when the stimuli were attended (solid red lines) vs. unattended (dashed red lines). Figure legend with line type and color coding associated with each experimental condition is given in title of (**A**,**B**) subplot rows.

Figure 2 presents sERP waveforms for four experimental conditions (ADSD, ADSV, AVSV, AVSD). Red lines are associated with the volar forearm surface stimulation (innervated with the median nerve) while blue lines are associated with the dorsal forearm surface stimulation (innervated with radial nerve). Colored dotted lines are responses to unattended hotspots while full lines are responses to attended hotpots. Figure 2 shows a general increase of sERP amplitude over multiple components induced with sustained spatial tactile attention task. This increase is registered for attending each of the stimulation hotspots. 

We have conducted a peak, latency search, and area under the curve calculation, for sERP of each subject within “search windows” presented in Table 1. Identified peaks and latencies for each condition and the results of their statistical comparison (*p* values) are given in Table 2. The values and statistics for differences between the areas under the voltage curves for each component are given in Table 3. 

**Figure 3:**
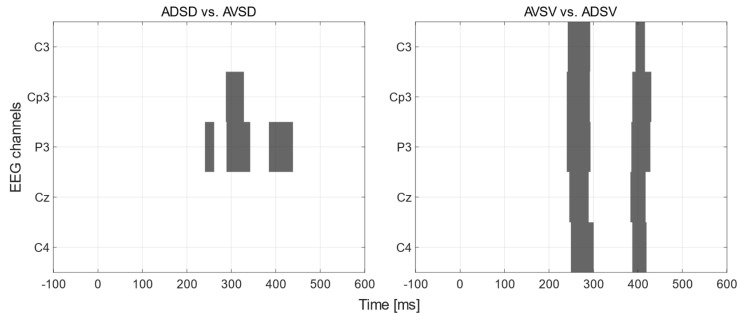
Intervals of statistical significance between conditions (gray color) and nonsignificant intervals (white color). The (**left**) graph shows the intervals for comparisons between ADSD and AVSD conditions and the (**right**) graph shows the intervals for comparisons between AVSV and ADSV conditions. The *y*-axis marks individual EEG channels and the *x*-axis time points in (*ms*), where 0 marks the stimulus onset. In all intervals found for both graphs the attended condition had a significantly higher amplitude than the unattended condition.

For the purpose of a deeper explorative analysis, comparisons between experimental conditions associated with the same stimulation hotspot, at all electrode sites, were conducted by using sERP amplitudes of consecutive time instants. Figure 3 shows the mapping of statistical significance between groups for certain time intervals. The interval of difference is considered significant only if statistically significant differences occurred in more than 12 consecutive time instants (corresponding to intervals of 10 *ms* duration or longer) to filter out statistical artifacts due to multiple comparisons, in line with the expected electrophysiological signal properties. Bold *p* values represent the statistically significant differences (*p* < 0.05). 

The obtained intervals of statistical significance between the attended and unattended conditions for the V stimulation location show two distinct intervals of significant differences, which are present in all channels and are temporally aligned. The first interval for V stimulation starts between 230 *ms* (P3) and 245 *ms* (C4) and ends between 288 *ms* (C3, CP3) and 304 *ms* (P3). The second interval for V stimulation starts between 381 *ms* (P3) and 388 *ms* (C4) and ends between 418 *ms* (C3) and 423 *ms* (C4). 

Regarding intervals of statistical significance between the attended and unattended conditions for D stimulation location, statistically significant differences were found only for CP3 and P3 channels. For the CP3 channel, a single interval (285 *ms*–307 *ms*) is present while for the P3 channel, three intervals (245 *ms*–256 *ms*, 290 *ms*–334 *ms* and 387 *ms*–435 *ms*) were found.

## 4. Discussion

This work introduced several methodological novelties in obtaining sERP responses. For this reason, the discussion of the obtained results and sERP components needs to address previous studies most comparable to our protocol design. Novelties introduced by our study design are:

(1) The stimulation of the mixed nerve branches instead of exclusively sensory nerve branches commonly used in basic ERP studies exploring tactile attention.
(2) The electrotactile attention task involving two stimulation hotspots with equal (50%) probability of stimulus occurrence, unlike commonly used oddball paradigm with rare and frequent stimulus.

Therefore, the obtained sERP morphology in our study is dependent on both novelties (the novel stimulation hotspot and the novel attention task paradigm). For this reason, in the next sections we discuss the obtained sERP responses for each stimulation hotspot independent of the attention task, to address the sERP morphology induced by the novel mixed nerve stimulation paradigm. Moreover, we discuss the effects of the sustained endogenous spatial attention task on the sERP features. Finally, we discuss the immediate implications of these findings for the design of electrotactile BCIs generally, and more specifically, the implications of the current results for further improvements in the control of our recently presented novel electrotactile BCI prototype [17]. 

### 4.1. Somatosensory ERP Morphology Associated with Mixed Nerve Stimulation

Studies of the brain mechanisms concerned with stimulus evaluation must differentiate between two classes of brain responses to any evoking sensory stimulus: (1) exogenous or obligatory responses which reflect the physiological registration of the sensory message in afferent pathways and cortical receiving areas, and (2) endogenous, cognitive components which reflect the activation of additional brain circuits in conjunction with the behavioral use of the information supplied by the sensory input [26].

In previous studies, somatosensory ERP responses elicited within the tactile attention task to electrical stimuli of exclusively sensory nerve branches of the fingers showed early (exogenous) components (N20, P40/50, N70) [14,27,28] which were unaffected by the cognitive (attention) task, and later components starting from 100 *ms* after the stimulus which were affected by the attention task (P100, N140, P300) [24,26,29]. In our study we applied somatosensory stimulation over the mixed nerve branches of median and radial nerves of the forearm by stimulating the innervated muscles (unlike stimulation of exclusively sensory nerve branches used in previous sERP research).

The morphology of the obtained somatosensory ERP components revealed distinct components (peaks) coinciding with the known morphology of P100, N140, P300 (specifically, the P3a–early P300 subcomponent, and P3b–late P300 subcomponent). We have not systematically extracted the components earlier than 100 *ms*, either because of subject specific responses to mixed nerve stimulation, or the contamination of early components by the stimulation artifact. However, the attention task explored in our work is expected to influence only the detected endogenous components as explained previously. Those components for each EEG channel are marked in grand average sERP curves (over all subjects and experimental conditions in Figure 1B). When sERP responses are grouped by stimulation location to waveforms reflecting the mixed median (V location) or mixed radial (D location) nerve, independent of the attention task (averaged over both attended and unattended conditions) the sERP morphology remains, showing the same distinct components for both stimulation hotspots (Figure 1A). The next section discusses the obtained effects of the attention task on each of the identified sERP components.

### 4.2. Effects of Endogenous Spatial Electrotactile Attention on Somatosensory ERP Components

#### 4.2.1. P100 Component

The P100 component is thought to reflect early sensory processing of the tactile stimulus, specifically the activation of primary somatosensory cortex in response to the tactile input [30]. Previous reports show that the amplitude of P100 increases in the attended condition [14,23,31,32].

In our study, statistical analysis of the P100 component amplitude, latency, or area under the voltage curve did not reveal any significant differences in any of the channels or stimulation locations. The grand average values of peak amplitudes reveal a trend of increase for the attended condition in all channels except C4 for D, and C3 and P3 for V. The grand average latency values for both stimulation locations and all channels show an increase in latency when stimulus is attended, however these differences didn’t reach statistical significance. These results imply a low sensitivity of P100 component features to our novel attention task/paradigm.

#### 4.2.2. N140 Component

N140 is defined as a prefrontal component that shows sensitivity to attention change [33]. Amplitude and latency of the N140 component are also modulated with attention shifts, while it is shown that the negative peak increases in amplitude when the stimulus is attended [31,34,35] to, whilst the latency decreases [36]. 

In our paradigm we detected the decrease of negativity of the N140 peak amplitude for all channels in the attended condition, for both stimulation locations. Average peak amplitude over all channels is −0.28 ± 1.24 *µV* (ADSD) vs. −0.94 ± 1.34 *µV* (AVSD), and 0.06 ± 1.20 *µV* (AVSV) vs. −0.71 ± 1.37 *µV* (ADSV). Statistically significant increases of amplitude in attended condition are shown for channels C3 and P3 for the D location, and in all channels except CP3 for the V location. These results are in contrast with related studies using the oddball paradigm to assess tactile attention task effects on somatosensory ERPs, [34,35], where attended stimuli were rare, with the occurrence probability of 0.2. Our study employed the same probability for attended and unattended stimuli (0.5), which may reflect the reverse effect on the N140 peak amplitude i.e., a decrease of N140 peak negativity instead of the increase detected with the oddball paradigm. Decrease in grand average latency for attended stimuli is visible on channels Cz, P3 (*p* < 0.05) and C4 (*p* < 0.05) for the D location (averaged over all channels 144.2 ± 23.6 *ms* for attended and 149.73 ± 29.34 *ms* for unattended condition), and for all channels except P3 for the V location (averaged over all channels 150.3 ± 26.9 *ms* for attended and 155.6 ± 26.2 *ms* for unattended condition). Area under the curve analysis showed statistically significant difference for channel P3 (D location) and channels C3, Cz, P3 and C4 (V), where the area under the curve decreased in attended condition.

#### 4.2.3. P3a and P3b Component

P3a and P3b are two distinct subcomponents of the P300 wave that occur in response to an external stimulus and are involved in stimulus evaluation or categorization. Both P3a and P3b are commonly studied in the context of cognitive and neural processing, and they have different functional and neural origins. P3a is an early subcomponent of the P300 ERP that typically occurs between 200–300 *ms* after a stimulus is presented. It is thought to reflect the automatic processing of novel or unexpected stimuli. P3b is a later subcomponent that occurs between 300–500 *ms* after a stimulus is presented. P3b is associated with the evaluation of the significance of a stimulus, memory retrieval, and decision-making processes. P3b is typically elicited by a more complex and meaningful stimulus, such as a target stimulus in a task where the participant is required to respond to it [37,38,39]. Our study revealed an increase in both components’ amplitudes for both stimulation locations in the attended condition which is in line with reports in previous studies on P300 associated with the tactile attention task. The presence of two distinct sERP components elicited by our novel paradigm with two equiprobable spatial stimuli of balanced amplitude may be attributed to the nature of our novel task. Namely, sequential presentation of randomized stimuli with 750 *ms* inter-stimulus intervals produces the novelty effect reflected in P3a, which is more pronounced in the attended condition (reflected in P3a amplitude increase), while the counting task of the attended stimuli reflected in the P3b presence may be attributed to the stimuli evaluation and memory processes (coinciding with P3b increase in attended condition). 

More specifically, our results show the increase of P3a peak amplitude in the attended condition for both stimulation locations (average value over all channels: 3.02 ± 1.87 *µV* (ADSD) vs. 1.69 ± 1.62 *µV* (AVSD), and 3.16 ± 1.12 *µV* (AVSV) vs. 2.20 ± 1.07 *µV* (ADSV). The statistical comparison showed significant differences in the peak amplitude for all channels for both stimulation locations, except for channel P3, the V location. Analysis of P3a latency did not result in statistical differences in any of the channels. A statistically significant increase of area under the P3a component in the attended condition is found in the Cz channel (D location), and for C3, Cp3, Cz and C4 channels (V location).

Statistical analysis of P3b component amplitude, latency, and areas under the curve, showed the most consistent differences between attended and unattended conditions. For both stimulation locations, all channels showed significant increase in peak amplitude for the attended condition (average peak amplitude over all channels: 2.73 ± 1.67 *µV* (ADSD) vs. 1.53 ± 1.40 *µV* (AVSD), and 3.23 ± 1.32 *µV* (AVSV) vs. 1.96 ± 1.50 *µV* (ADSV)). Area under the P3b peak proved to be the most consistent index since it gave statistically significant differences for all channels, and for both V and D locations.

The obtained intervals of statistical significance (Figure 3) between the attended and unattended condition and V stimulation location showed two distinct intervals of significant differences, temporally aligned over all channels, while for the D stimulation location statistically significant differences were found only for CP3 and P3 channels. The found intervals coincide with the timings of P3a and P3b components and show the distinct regions of temporal mismatch created by the sustained spatial electrotactile attention task.

### 4.3. Prospects for Tactile BCI Applications

Tactile brain-computer interfaces (BCIs) are a relatively new and emerging field of research, and as such, they have received less attention than other types of BCIs, such as visual, auditory, or motor BCIs [40,41,42,43]. Tactile BCIs are the least studied among all stimulus dependent (reactive) BCIs, probably due to the technical complexity of tactile stimulators compared to visual or auditory stimulation hardware. Tactile stimulators are less widely available and can be more difficult to integrate into BCI systems [44]. However, there are specific needs and challenges in the BCI field that can be addressed by the introduction of tactile modality in BCI control. For example, most visual BCIs are based on presenting several flickering stimuli to the user either from a computer screen or a custom stimulation device, and the continuous flickering can cause visual fatigue and reduce users’ comfort [45]. Moreover, unlike most visual BCIs that are to some extent gaze dependent, the tactile BCIs can be completely gaze independent [46]. Tactile interfaces can be useful for individuals who are visually or auditory impaired, in situations where visual or auditory information is overwhelming or distracting and in tasks that require spatial awareness, such as navigation or manipulation of objects [47,48,49]. Tactile interfaces can be more private than visual or auditory interfaces, can be concealed and wearable [50]. From the perspective of BCI application in the domain of neurofeedback and neuromodulation, the tactile interfaces may be applicable for sensorimotor neuromodulation to induce neuroplasticity, since the BCI control modality directly interacts with the sensorimotor network [13]. The current study explores a completely novel BCI control signal, the somatosensory ERP, elicited by stimulation of two neighboring hotspots on the same upper limb. Moreover, unlike most previously explored somatosensory evoked and event-related potentials where the stimulation hotspots were placed over the exclusively sensory nerve branches, our sERP signals were induced by the stimulation of mixed nerves. Here we presented the morphology of sERP responses induced by the stimulation of two different branches of mixed nerves by stimulating the innervated muscles below the sensory threshold. The obtained results confirmed that the morphology of the sERP responses expressed the same ERP components when comparing the responses associated with 2 stimulation hotspots (V and D) and that those components are in the same latency ranges as the main endogenous ERP components registered previously when stimulating exclusively sensory nerves. The effects of the sustained endogenous spatial electrotactile attention task on the sERP obtained for both stimulation hotspots were in line with the previous reports [22,26,51]. Namely, we detected an overall increase of ERP amplitude during the attended condition. It’s important to note that the sERP induced by our task by the sustained spatial electrotactile attention involved two stimulation hotspots with equal (50%) probability of stimulus occurrence. Therefore, our presented sERP responses correspond to predominantly endogenous spatial tactile attention. Presented sERPs are completely independent of target/distractor probability, unlike in the vast majority of ERP-based BCIs utilizing the oddball paradigm with rare and frequent stimuli [52]. 

The limitation of the current study is the lower number of recorded EEG channels which prevents a more comprehensive spatial analysis of the obtained sERP components. This limitation should be addressed in future basic studies exploring the effects of endogenous spatial tactile attention on sERP. The selection of the EEG channels in our current study was constrained to the analysis of sensorimotor areas since the effects of our recently presented electrotactile BCI for sensory training [17] are expected to be reflected in the reorganization of sensorimotor cortex. The lower number of channels was in line with intended applications in tactile BCIs where the control paradigms based on smaller subset of channels are favorable.

Future work will involve testing the identified features of N140, P3a, and P3b in the online classification of the endogenous spatial tactile attention. Moreover, this paradigm may be utilized both for sensory training in the stroke population and possibly as a communication channel in the ALS population.

## 5. Conclusions

The results of this study are the first to show that the sERP morphology associated with a novel sustained spatial electrotactile attention task, first introduced in our recent work exploring electrotactile BCI for sensory training [17]. Our results on the sERP components when stimulating mixed nerve branches revealed that the ERP morphology is similar to previous reports on components obtained by the stimulation of exclusively sensory nerves, i.e., we validated the presence of P100, N140, P3a, and P3b peaks for mixed radial and median nerve stimulation. Our novel task for estimating the effects of endogenous spatial tactile attention using two equiprobable tactile stimuli induced a general increase in the sERP amplitude, reaching statistical significance in N140, P3a, and P3b components. These results further support the possibility of the use of mixed nerves sERP, proximal stimulation hotspots, and equal (50%) probability of target/distractor stimuli in sERP-based BCI designs. The results show that features of the P3b, P3a, and N140 components are most prominent global markers of sustained spatial electrotactile attention over all subjects, and this work proposes that the features of those components (peak amplitudes, latencies, and areas under the voltage curves) are potential features for online BCI control.

## Figures and Tables

**Table 1:** Identified ERP components from the grand average somatosensory ERP morphology, and search windows (poststimulus intervals in milliseconds) used for the measurement of peak amplitudes, peak latencies, and areas under the ERP waveforms.

sERP Component	Search Window [*ms*]
P100	60–140
N140	110–200
P3a	200–280
P3b	280–400

**Table 2:** Values of grand average peak amplitudes and peak latencies for each EEG channel, sERP component (P100, N140, P3a and P3b) and condition (ADSD, AVSD, AVSV, ADSV) are included. *p* values of statistical comparisons between attended and unattended conditions associated with locations D and V (ADSD vs. AVSD and AVSV vs. ADSV) are included in columns Statistics D and Statistics V, respectively.

	Channel		ADSD	AVSD	Statistics D	AVSV	ADSV	Statistics V
**P100 component**	C3	Peak [*µV*]	1.47 ± 1.33	1.14 ± 1.21	*p* = 0.244	1.68 ± 0.79	1.56 ± 0.88	*p* = 0.685
		Latency [*ms*]	101.21 ± 28.95	100.23 ± 29.74	*p* = 0.520	102.35 ± 32.18	95.61 ± 29.63	*p* = 0.148
	CP3	Peak [*µV*]	2.15 ± 1.08	1.88 ± 1.34	*p* = 0.273	1.99 ± 1.12	2.15 ± 0.72	*p* = 0.414
		Latency [*ms*]	91.21 ± 27.35	84.39 ± 23.56	*p* = 0.157	90.61 ± 26.53	83.71 ± 24.30	*p* = 0.201
	P3	Peak [*µV*]	2.29 ± 1.65	1.88 ± 1.44	*p* = 0.340	2.79 ± 1.22	2.32 ± 1.06	*p* = 0.273
		Latency [*ms*]	121.52 ± 21.35	117.73 ± 27.58	*p* = 0.910	121.82 ± 22.70	121.52 ± 26.23	*p* = 0.678
	Cz	Peak [*µV*]	2.26 ± 1.33	1.91 ± 1.63	*p* = 0.191	1.99 ± 1.33	2.14 ± 1.02	*p* = 0.497
		Latency [*ms*]	84.92 ± 22.66	77.73 ± 16.85	*p* = 0.096	86.74 ± 21.66	82.27 ± 17.15	*p* = 0.850
	C4	Peak [*µV*]	1.22 ± 1.37	1.26 ± 1.19	*p* = 0.839	1.54 ± 0.87	1.57 ± 0.73	*p* = 1.000
		Latency [*ms*]	108.33 ± 20.90	105.83 ± 19.17	*p* = 0.985	106.29 ± 25.65	99.24 ± 23.63	*p* = 0.413
**N140 component**	C3	Peak [*µV*]	−0.14 ± 1.37	−0.80 ± 1.31	***p* = 0.048**	−0.04 ± 1.29	−0.69 ± 1.59	***p* = 0.048**
		Latency [*ms*]	140.08 ± 21.73	139.92 ± 27.66	p = 0.904	138.94 ± 27.00	146.06 ± 28.44	p = 0.168
	CP3	Peak [*µV*]	−0.73 ± 1.23	−1.38 ± 1.40	p = 0.080	−0.46 ± 1.17	−0.95 ± 1.30	p = 0.057
		Latency [*ms*]	139.70 ± 22.61	139.32 ± 25.39	p = 0.814	142.80 ± 25.59	149.92 ± 24.07	p = 0.339
	P3	Peak [*µV*]	0.62 ± 1.45	−0.29 ± 1.41	***p* = 0.006**	1.23 ± 1.38	−0.09 ± 1.55	***p* = 0.002**
		Latency [*ms*]	143.64 ± 25.20	154.47 ± 33.85	***p* = 0.037**	150.00 ± 30.97	161.06 ± 29.81	*p* = 0.492
	Cz	Peak [*µV*]	−0.79 ± 1.12	−1.35 ± 1.54	p = 0.080	−0.65 ± 1.24	−1.11 ± 1.20	***p* = 0.040**
		Latency [*ms*]	149.09 ± 24.10	154.55 ± 30.57	p = 0.227	157.42 ± 26.25	153.56 ± 22.50	*p* = 0.765
	C4	Peak [*µV*]	−0.34 ± 1.04	−0.88 ± 1.04	p = 0.094	0.19 ± 0.92	−0.70 ± 1.19	***p* = 0.008**
		Latency [*ms*]	148.48 ± 24.53	160.38 ± 29.22	***p* = 0.020**	162.50 ± 24.74	167.50 ± 26.43	*p* = 0.276
**P3a component**	C3	Peak [*µV*]	3.44 ± 1.94	2.21 ± 1.82	***p* = 0.002**	3.57 ± 1.24	2.57 ± 1.09	***p* = 0.002**
		Latency [*ms*]	243.86 ± 24.51	239.70 ± 24.92	*p* = 0.128	257.65 ± 17.22	252.12 ± 28.89	*p* = 0.918
	CP3	Peak [*µV*]	2.82 ± 1.75	1.59 ± 1.45	***p* = 0.003**	2.96 ± 0.90	2.16 ± 0.88	***p* = 0.033**
		Latency [*ms*]	249.47 ± 21.67	240.00 ± 26.78	*p* = 0.182	261.82 ± 19.65	252.50 ± 29.58	*p* = 0.359
	P3	Peak [*µV*]	4.14 ± 2.48	2.53 ± 2.24	***p* = 0.001**	4.30 ± 1.73	2.88 ± 1.61	***p* = 0.000**
		Latency [*ms*]	246.29 ± 23.81	245.91 ± 23.90	*p* = 1.000	260.30 ± 19.35	252.27 ± 30.08	*p* = 0.496
	Cz	Peak [*µV*]	2.18 ± 1.61	0.78 ± 1.25	***p* = 0.003**	2.16 ± 0.70	1.55 ± 0.73	*p* = 0.168
		Latency [*ms*]	251.52 ± 22.90	239.85 ± 29.51	*p* = 0.278	261.59 ± 20.60	252.12 ± 30.79	*p* = 0.383
	C4	Peak [*µV*]	2.48 ± 1.56	1.37 ± 1.35	***p* = 0.017**	2.79 ± 1.06	1.83 ± 1.05	***p* = 0.003**
		Latency [*ms*]	244.17 ± 22.47	232.58 ± 27.57	*p* = 0.185	262.05 ± 20.10	250.98 ± 31.26	*p* = 0.164
**P3b component**	C3	Peak [*µV*]	3.18 ± 2.00	1.98 ± 1.53	***p*= 0.002**	3.52 ± 1.52	2.34 ± 1.63	***p* = 0.001**
		Latency [*ms*]	318.79 ± 26.30	333.71 ± 36.45	**p = 0.014**	319.24 ± 39.40	318.11 ± 33.16	*p* = 0.922
	CP3	Peak [*µV*]	2.75 ± 1.61	1.42 ± 1.36	***p* = 0.001**	3.05 ± 1.26	1.73 ± 1.28	***p* = 0.001**
		Latency [*ms*]	315.76 ± 22.81	332.65 ± 35.17	***p* = 0.007**	318.79 ± 41.63	326.59 ± 33.71	*p* = 0.677
	P3	Peak [*µV*]	3.34 ± 1.98	2.02 ± 1.53	***p* = 0.001**	3.98 ± 1.53	2.57 ± 1.73	***p* = 0.002**
		Latency [*ms*]	310.68 ± 25.22	324.62 ± 37.09	***p* = 0.006**	325.45 ± 46.39	327.20 ± 33.06	*p* = 0.850
	Cz	Peak [*µV*]	2.27 ± 1.27	0.88 ± 1.27	***p* = 0.002**	2.49 ± 1.01	1.18 ± 1.14	***p* = 0.001**
		Latency [*ms*]	328.86 ± 26.24	337.73 ± 39.11	*p* = 0.349	341.97 ± 34.71	327.80 ± 34.20	*p* = 0.077
	C4	Peak [*µV*]	2.10 ± 1.50	1.37 ± 1.30	***p* = 0.027**	3.09 ± 1.29	1.98 ± 1.73	***p* = 0.008**
		Latency [*ms*]	335.00 ± 31.20	339.47 ± 39.57	*p* = 0.233	343.71 ± 37.24	345.23 ± 37.16	*p* = 0.866

**Table 3:** The values of grand average areas under the voltage curves for each EEG channel, sERP component (P100, N140, P3a and P3b) and condition (ADSD, AVSD, AVSV, ADSV) are included. *P* values of statistical comparisons between attended and unattended conditions associated with locations D and V (ADSD vs. AVSD and AVSV vs. ADSV) are included in columns Statistics D and Statistics V, respectively. Bold *p* values represent the statistically significant differences (*p* < 0.05).

	Channel		ADSD	AVSD	Statistics D	AVSV	ADSV	Statistics V
**P100 component**	C3	Area [ms⋅μV]	0.10 ± 0.05	0.08 ± 0.04	*p* = 0.216	0.08 ± 0.03	0.09 ± 0.04	*p* = 0.635
	CP3		0.11 ± 0.03	0.11 ± 0.04	*p* = 0.893	0.10 ± 0.04	0.10 ± 0.04	*p* = 0.787
	P3		0.13 ± 0.09	0.12 ± 0.06	*p* = 0.839	0.13 ± 0.07	0.11 ± 0.06	*p* = 0.216
	Cz		0.11 ± 0.05	0.12 ± 0.05	*p* = 0.414	0.11 ± 0.05	0.10 ± 0.05	*p* = 0.414
	C4		0.09 ± 0.06	0.09 ± 0.05	*p* = 1.000	0.10 ± 0.04	0.08 ± 0.04	*p* = 0.168
**N140 component**	C3	Area [ms⋅μV]	0.11 ± 0.07	0.10 ± 0.05	*p* = 0.146	0.11 ± 0.08	0.11 ± 0.07	***p* = 0.040**
	CP3		0.09 ± 0.04	0.09 ± 0.06	*p* = 0.305	0.08 ± 0.06	0.10 ± 0.06	*p* = 0.080
	P3		0.15 ± 0.10	0.12 ± 0.08	***p* = 0.040**	0.18 ± 0.11	0.14 ± 0.07	***p* = 0.005**
	Cz		0.08 ± 0.05	0.09 ± 0.06	*p* = 0.168	0.08 ± 0.04	0.08 ± 0.05	***p* = 0.048**
	C4		0.08 ± 0.05	0.07 ± 0.03	*p* = 0.414	0.08 ± 0.05	0.08 ± 0.04	***p* = 0.010**
**P3a component**	C3	Area [ms⋅μV]	0.21 ± 0.14	0.16 ± 0.10	*p* = 0.080	0.22 ± 0.08	0.14 ± 0.07	***p* = 0.002**
	CP3		0.17 ± 0.13	0.12 ± 0.08	*p* = 0.080	0.17 ± 0.05	0.11 ± 0.05	***p* = 0.008**
	P3		0.25 ± 0.17	0.16 ± 0.13	***p* = 0.000**	0.26 ± 0.10	0.16 ± 0.09	***p* = 0.000**
	Cz		0.13 ± 0.10	0.09 ± 0.06	*p* = 0.094	0.11 ± 0.05	0.08 ± 0.04	*p* = 0.080
	C4		0.14 ± 0.10	0.09 ± 0.07	*p* = 0.080	0.15 ± 0.06	0.12 ± 0.04	***p* = 0.021**
**P3b component**	C3	Area [ms⋅μV]	0.26 ± 0.23	0.17 ± 0.13	***p* = 0.006**	0.32 ± 0.19	0.22 ± 0.17	***p* = 0.001**
	CP3		0.22 ± 0.18	0.15 ± 0.09	***p* = 0.017**	0.27 ± 0.15	0.18 ± 0.10	***p* = 0.002**
	P3		0.27 ± 0.23	0.16 ± 0.11	***p* = 0.021**	0.36 ± 0.19	0.24 ± 0.16	***p* = 0.002**
	Cz		0.18 ± 0.13	0.12 ± 0.08	***p* = 0.040**	0.22 ± 0.11	0.14 ± 0.08	***p* = 0.021**
	C4		0.18 ± 0.17	0.12 ± 0.09	***p* = 0.048**	0.28 ± 0.16	0.18 ± 0.15	***p* = 0.005**

## Data Availability

The data that support the findings of this study will be made available by the corresponding author A.M.S., upon reasonable request.
